# Supplementary material for: Hybrid Models and Biological Model Reduction with PyDSTool
Source: PLoS Comput Biol. 2012 Aug 9;8(8):e1002628. doi: 10.1371/journal.pcbi.1002628 (PMC3415397; doi:10.1371/journal.pcbi.1002628)
Supplement: Text S4 — Complete source code for the PyDSTool package (version 0.88.120504). Includes API documentation and help files linking to web pages. This file is identical to the current public release on Sourceforge.net. (ZIP) [file pcbi.1002628.s004.zip › PyDSTool/html/PyDSTool.Toolbox.PySCes_SBML-module.html]

xml version="1.0" encoding="ascii"?


PyDSTool.Toolbox.PySCes\_SBML


| Home | Trees | Indices | Help | PyDSTool |
| --- | --- | --- | --- | --- |

Package PyDSTool ::
Package Toolbox ::
Module PySCes\_SBML

[hide private]

[frames] | no frames]

# Module PySCes\_SBML

source code

PySCes interface code for systems biology modeling and SBML model
markup.

This toolbox assumes you have PySCes installed.

R. Clewley, 2012


|  |  |
| --- | --- |
| Functions | [hide private] |

|  |  |
| --- | --- |
| make\_varspecs(m, fnspecs) | source code |

|  |  |
| --- | --- |
| get\_pysces\_model(filename, target=`'``Vode``'`) | source code |


|  |  |
| --- | --- |
| Variables | [hide private] |

\_seq\_types = `(<type 'list'>, <type 'tuple'>, <type 'numpy.ndar...`

\_num\_types = `(<type 'float'>, <type 'int'>, <type 'numpy.float...`

isfinite = `<ufunc 'isfinite'>`

sign = `<ufunc 'sign'>`

\_functions = `['get_pysces_model']`

\_classes = `[]`

\_features = `[]`


|  |  |
| --- | --- |
| Variables Details | [hide private] |

### \_seq\_types

Value:
:   ```
    (<type 'list'>, <type 'tuple'>, <type 'numpy.ndarray'>)
    ```

### \_num\_types

Value:
:   ```
    (<type 'float'>,
     <type 'int'>,
     <type 'numpy.floating'>,
     <type 'numpy.integer'>)
    ```

  


| Home | Trees | Indices | Help | PyDSTool |
| --- | --- | --- | --- | --- |

|  |  |
| --- | --- |
| Generated by Epydoc 3.0.1 on Fri May 4 15:24:04 2012 | http://epydoc.sourceforge.net |
